# Advancing brain-inspired computing with hybrid neural networks

**DOI:** 10.1093/nsr/nwae066

**Published:** 2024-02-26

**Authors:** Faqiang Liu, Hao Zheng, Songchen Ma, Weihao Zhang, Xue Liu, Yansong Chua, Luping Shi, Rong Zhao

**Affiliations:** Center for Brain-Inspired Computing Research, Optical Memory National Engineering Research Center, Tsinghua University-China Electronics Technology HIK Group Co. Joint Research Center for Brain-inspired Computing, IDG/McGovern Institute for Brain Research, Department of Precision Instrument, Tsinghua University, Beijing 100084, China; Center for Brain-Inspired Computing Research, Optical Memory National Engineering Research Center, Tsinghua University-China Electronics Technology HIK Group Co. Joint Research Center for Brain-inspired Computing, IDG/McGovern Institute for Brain Research, Department of Precision Instrument, Tsinghua University, Beijing 100084, China; Center for Brain-Inspired Computing Research, Optical Memory National Engineering Research Center, Tsinghua University-China Electronics Technology HIK Group Co. Joint Research Center for Brain-inspired Computing, IDG/McGovern Institute for Brain Research, Department of Precision Instrument, Tsinghua University, Beijing 100084, China; Center for Brain-Inspired Computing Research, Optical Memory National Engineering Research Center, Tsinghua University-China Electronics Technology HIK Group Co. Joint Research Center for Brain-inspired Computing, IDG/McGovern Institute for Brain Research, Department of Precision Instrument, Tsinghua University, Beijing 100084, China; Center for Brain-Inspired Computing Research, Optical Memory National Engineering Research Center, Tsinghua University-China Electronics Technology HIK Group Co. Joint Research Center for Brain-inspired Computing, IDG/McGovern Institute for Brain Research, Department of Precision Instrument, Tsinghua University, Beijing 100084, China; Neuromorphic Computing Laboratory, China Nanhu Academy of Electronics and Information Technology, Jiaxing 314001, China; Center for Brain-Inspired Computing Research, Optical Memory National Engineering Research Center, Tsinghua University-China Electronics Technology HIK Group Co. Joint Research Center for Brain-inspired Computing, IDG/McGovern Institute for Brain Research, Department of Precision Instrument, Tsinghua University, Beijing 100084, China; Center for Brain-Inspired Computing Research, Optical Memory National Engineering Research Center, Tsinghua University-China Electronics Technology HIK Group Co. Joint Research Center for Brain-inspired Computing, IDG/McGovern Institute for Brain Research, Department of Precision Instrument, Tsinghua University, Beijing 100084, China

**Keywords:** brain-inspired computing, hybrid neural network, dual-brain driven, multi-network integration, neuromorphic system

## Abstract

Brain-inspired computing, drawing inspiration from the fundamental structure and information-processing mechanisms of the human brain, has gained significant momentum in recent years. It has emerged as a research paradigm centered on brain–computer dual-driven and multi-network integration. One noteworthy instance of this paradigm is the hybrid neural network (HNN), which integrates computer-science-oriented artificial neural networks (ANNs) with neuroscience-oriented spiking neural networks (SNNs). HNNs exhibit distinct advantages in various intelligent tasks, including perception, cognition and learning. This paper presents a comprehensive review of HNNs with an emphasis on their origin, concepts, biological perspective, construction framework and supporting systems. Furthermore, insights and suggestions for potential research directions are provided aiming to propel the advancement of the HNN paradigm.

## Teaser

Discover the fascinating world of hybrid neural networks revolutionizing artificial intelligence by gaining insights into their origin, construction framework, and supporting systems, along with potential future research directions.

## INTRODUCTION

The human brain stands out as the sole biological organism exhibiting advanced general intelligence with ultra-low power consumption. Leveraging insights from the brain holds the potential to propel the development of narrow artificial intelligence towards the realm of artificial general intelligence (AGI) [1,2]. Embracing this philosophy, brain-inspired computing (BIC) introduces a novel paradigm for computing and learning inspired by the fundamental structures and information-processing mechanisms of the human brain. The BIC system encompasses a diverse array of components including hardware, software, models and algorithms, which necessitates collaborations across various disciplines such as psychology, mathematics, neuroscience, material science, microelectronics and computer science.

The development of BIC has progressed through four significant stages, marked by the enrichment of its concepts and paradigm shifts in research focus. The initial milestone occurred in the late 1980s when the concept of neuromorphic computing was introduced, primarily focusing on emulating biological neurons [3], retina [4] and cochlea [5]. During this stage, BIC was aimed at leveraging highly parallel analog circuits to achieve ultra-low power consumption. Advancements primarily concentrated on hardware innovations, leading to a continuous improvement in the fidelity of emulated neural organisms. Neuromorphic sensors and computing circuits emerged successively.

The second stage occurred around the 2000s when spiking neural networks (SNNs) and their corresponding training algorithms, emphasizing biological fidelity [6–8], experienced rapid development. Meanwhile, brain-inspired visual and auditory sensors [9–11] developed rapidly, offering a more suitable data format for BIC networks and presenting notable advantages over traditional sensors in terms of power consumption, dynamic range and data rate. During this phase, a primary application of BIC was to simulate the brain through numerical calculations.

The third critical milestone took place around the 2010s, characterized by the emergence of SNNs with machine-learning capabilities [12–15], which demonstrated remarkable performance in various intelligent tasks, such as image classification [16] and voice recognition [17]. Concurrently, the field of chip-level neuromorphic computing hardware made substantial progress, leading to the emergence of highly integrated BIC chips [18–23]. Notably, there was a synergistic development of BIC chips and models, which not only advanced the research on brain simulation, but also facilitated practical industrial applications.

The fourth key milestone occurred in 2019 with the introduction of the Tianjic [2] BIC chip that embodies the capability to support both computer-science-oriented models and neuroscience-inspired models, engendering a new pathway for the development of AGI systems in a synergistic approach. In particular, the Tianjic platform provides a hybrid architecture proficient in seamlessly supporting both artificial neural networks (ANNs) and SNNs. This establishes a dual-brain-driven computing paradigm, enabling the realization and utilization of hybrid neural networks (HNNs). Since then, HNNs have garnered considerable research interest, driving their rapid development and diverse applications, characterized by unique advantages in various intelligent tasks, such as perception, cognition and learning [2,24].

This review provides a comprehensive review of HNNs from multiple perspectives, including their biological underpinnings, construction framework, chip-level implementation, software and system infrastructure. Moreover, promising research directions for future research and exploration of HNNs are discussed.

## CONCEPT OF HNNs

**Figure 1.**
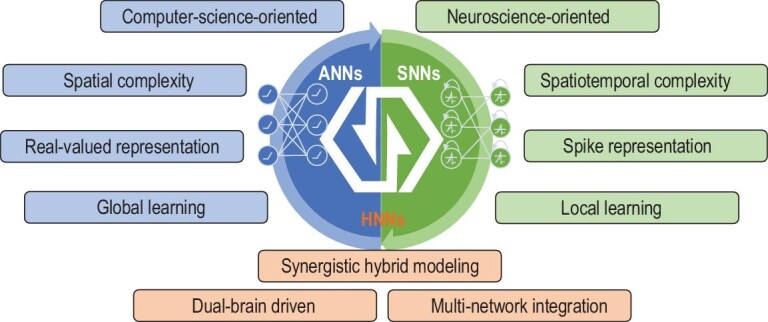
The concept of HNNs. HNNs combine key features of computer-science-oriented models and neuroscience-oriented models, demonstrating improved flexibility and universality in supporting advanced intelligence.

HNNs are instances of the dual-brain-driven paradigm that integrates neuroscience-oriented networks and computer-science-oriented networks in a comprehensive manner, resulting in a holistic framework showcasing distinct attributes from different perspectives including computing paradigm, multi-network integration, signal representation and information processing, as illustrated in Fig. 1. Computer-science-oriented models, represented by ANNs featuring dense and precise computation, possess characteristics such as spatial complexity and general approximation ability. Especially, ANNs capture the integration and transformation of features in biological neurons at a high level. In contrast, neuroscience-oriented models, represented by SNNs [25,26] driven by neural dynamics, incorporate architectures of brain neurons and network and processing mechanisms in a comprehensive and multi-granular manner. Therefore, SNNs exhibit unique attributes such as state memory, threshold switching and diverse coding schemes, demonstrating strong spatio-temporal information-processing capabilities [16,27,28]. Table 1 provides an elucidation of the key features of ANNs, SNNs and brain networks. HNNs effectively leverage the merits of models from both paradigms under appropriate conditions, thereby offering the potential to facilitate the development of AGI. To a certain extent, analogous to the role of p-type and n-type semiconductors forming PN junctions for transistors in the hardware of modern information systems, the integration of ANNs and SNNs to create HNNs serves as a basic building block in the development of advanced AGI systems.

**Table 1.** Key features of ANNs, SNNs and brain networks.

	ANNs	SNNs	Brain networks
Basic model	Mainly based on a static soma model composed of linear accumulation and non-linear activation	Multi-compartmental neuron model with temporal dynamics and dendrite computation	Complex biological structures with diverse biochemical reactions and signaling mechanisms
Connection types	Mainly composed of forward dense connections such as fully connected and convolutional connection	Various connection types such as forward connection, lateral connection and feedback with high sparsity	Forward, feedback and lateral connections with ultra-high sparsity, and synaptic growth and elimination mechanisms
Network architecture	Relatively regular and single structure, such as cascaded convolutional and fully connected layers	Having structures similar to ANNs, as well as richer structures inspired by the brain networks	Small-world topology, scale-free connectivity, modular organization, reciprocal connections, functional hierarchy, etc.
Coding schemes	Mainly based on rate coding	Various coding schemes such as rating coding, temporal coding and population coding	Various multiscale coding schemes
Learning algorithms	Mainly based on global end-to-end learning by gradient descent	Local learning such as spike-timing-dependent plasticity and global learning such as gradient descent	Various multiscale, multi-granularity learning mechanisms
Computing capability	Mainly based on spatial complexity	Spatio-temporal complexity	Spatio-temporal complexity
Power consumption	High power consumption	Low power consumption	Ultra-low power consumption
Synchronism	Synchronized	Synchronized and asynchronized	Synchronized and asynchronized
Skilled tasks	Deterministic, static, specific tasks in noise-free environments	Dynamic, sequential tasks in noisy environments	Dynamic tasks in the noisy open world

The multi-network integration aspect of HNNs offers a wider range of building blocks, enabling greater flexibility and diverse functionalities. Particularly in the current era of large foundation models [29,30], HNNs introduce a new dimension and methodology to enhance the capabilities of intelligent models. This can be achieved by integrating multiple pre-trained networks with different attributes across various aspects, establishing prerequisites that enable HNNs to effectively exploit their integration advantages. Furthermore, HNNs offer a viable way to tackle challenges associated with extensive computational demands, storage requirements and data collection that arise when training a single large end-to-end model from scratch.

In this review paper, two significant heterogeneities within HNNs are presented and analysed to enhance integration: the distinct signal representation and information-processing characteristics of ANNs and SNNs. Regarding signal representation, SNNs utilize binary spike trains to encode information and rely heavily on the temporal dimension, as indicated by the continuous value of the spike emission time. In contrast, ANNs rely on the magnitudes of continuous values or vectors for information encoding. When dealing with sequential data, ANNs typically process real-valued sequences with uniform time intervals. Exploiting the variance in representation precision between ANNs and SNNs can be efficiently harnessed in hardware implementation to strike a balance between performance and cost. For instance, analog and asynchronous circuits find suitability in accommodating SNNs, while synchronous digital circuits are more suitable for ANNs.

From an information-processing perspective, ANNs primarily depend on single feedforward processing and spatial complexity resulting from neuronal connections. On the other hand, SNNs leverage spatio-temporal complexity to solve problems through multiple iterations. While temporal and spatial processing can often be interchanged, it is noteworthy that multistep temporal iteration has the potential to mitigate overall complexity by reusing intermediate results, akin to dynamic programming. The ability to reuse intermediate states stands as a distinct advantage of spatio-temporal complexity.

As fusion models, HNNs possess unique characteristics that allow adaptive selection of base models based on the specific requirements, resulting in a synergistic effect. At the algorithm level, compared with single ANNs and single SNNs, HNNs can achieve a much better balance of comprehensive performance [24]. In certain scenarios, HNNs can produce a synergy effect of 1 + 1 > 2 [24]. On the other hand, HNNs, composed of heterogeneous ANNs and SNNs, greatly enhance the design space, making it easier to implement adaptive design strategies. At the implementation level, the Tianjic series of chips [2,31] and corresponding software hierarchy [32] have undergone systematic optimization in terms of computing units, storage structures and communication facilities to support efficient operations of HNNs. This enables the deployment and execution of HNNs to be highly efficient, providing the possibility to achieve excellent comprehensive performance balance and a broad design space for HNNs.

HNNs share similarities with ensemble models [33] or MoE (mixture of experts) models [34], both of which aim to enhance performance by integrating multiple models. However, HNNs exhibit significant differences from these models. In terms of their basic components, HNNs integrate neurons, modules and networks from both ANNs and SNNs. This unique combination has not been extensively studied or utilized in other ensemble learning models or models such as MoE, and thus presents a promising research space for exploration. Regarding the construction methods, the integration of ANNs and SNNs necessitates addressing the compatibility issue of heterogeneous signals and representations. Moreover, compared with ensemble learning models, the construction methods of HNNs involve comprehensive considerations of integration paradigms, information flow, interaction modes and topological structures. Consequently, the integration of ANNs and SNNs in HNNs brings about unique heterogeneity. This not only presents challenges such as compatibility, which are not encountered in general ensemble learning models, but also provides a greater design space and other distinctive advantages for fusion.

**Figure 2.**
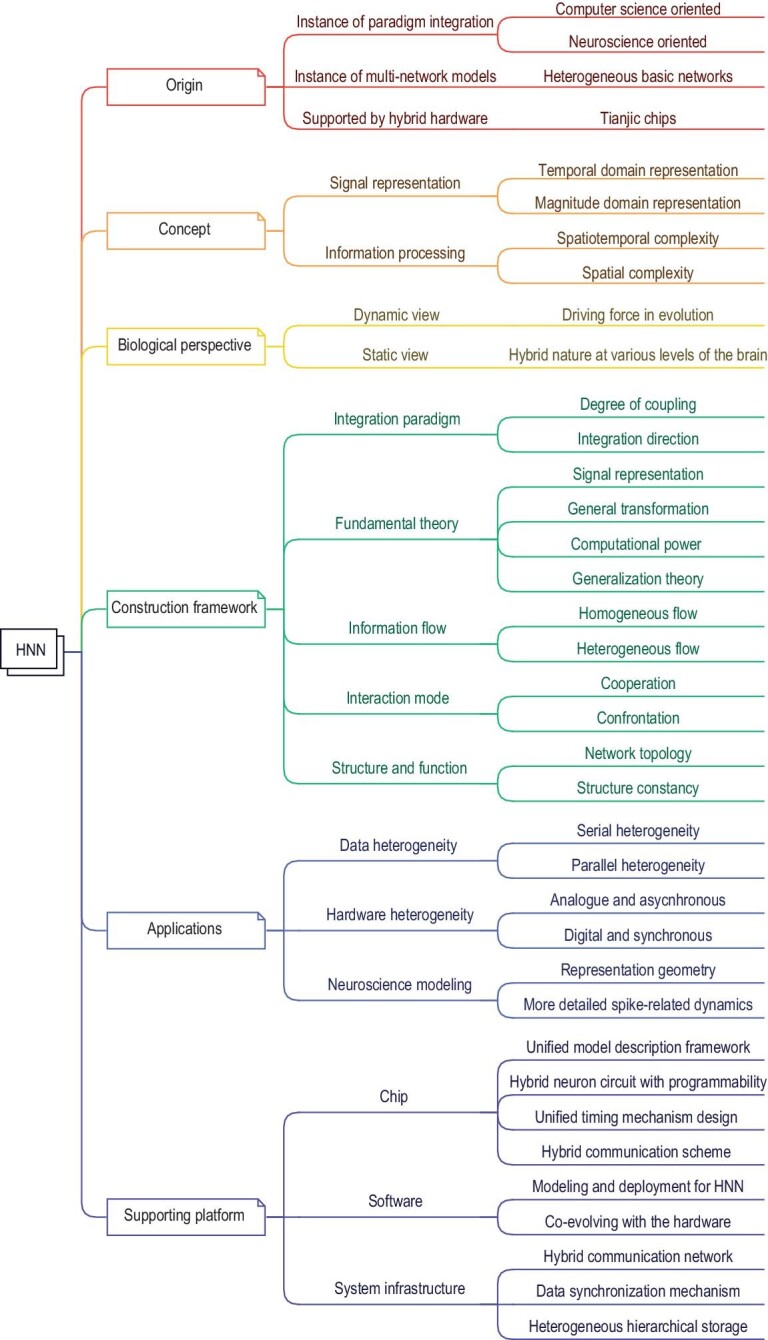
The landscape of HNNs. The landscape includes the origin, concept, biological perspective, construction framework, chip, software and system infrastructure of HNNs.

Figure 2 provides a comprehensive landscape diagram of HNNs, covering various aspects from their origin, concepts, biological perspectives, applications, construction frameworks and supporting platforms including chip implementation, software and system infrastructure. The following sections delve into these aspects in greater detail.

## A BROAD BIOLOGICAL PERSPECTIVE OF HNNs

**Figure 3.**
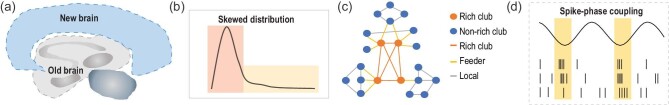
A broad biological perspective of HNNs. (a) Evolution of the neocortex. (b) Skewed distribution in the brain. (c) Rich-club organization in the brain. (d) Spike-phase coupling in the brain.

The origins and advantages of HNNs can be explored from a biological perspective. Biological evolution, where intelligence emerges, can be seen as a hybridization process (Fig. 3). For instance, the neocortex—a more recently developed brain region responsible for higher-order functions—built upon old brain regions directed for survival needs [35] (Fig. 3a). In essence, a general driving force for evolution within the vast array of diverse possible biological configurations is the hybridization between different lineages, which often generates synergistic competitive advantages. The older mechanisms persist, but they serve at different levels, with new adaptations emerging along the evolution process. The HNN is an instance of such hybrid integration inspired by the hybridization process in biology, with various heterogeneous characteristics in multiple aspects.

The evolved biological brain intrinsically exhibits heterogeneity. At the structural level, as proposed by Buzaki's inegalitarian log-scale brain theory [36], most observable quantities in the nervous system, such as the firing rate, axonal length and width, spine size, time constant, etc., follow skewed distributions (Fig. 3b). This distribution spans a continuous spectrum of heterogeneity with a long tail across several spatial-temporal scales. Moreover, these quantities induce qualitative changes in dynamical properties, leading to two discrete classes of substrates underlining the distribution: a core rich-club network [37] (Fig. 3c) with a minority of neurons (20%) and a peripheral subnetwork with a majority of neurons (80%). The hybrid of these two subsystems enables the essential balance between accuracy and speed, as well as stability, and plasticity of the brain [38]. At the functional level, the hybrid interaction among multiple heterogeneous brain modules is evident in the various couplings and synchronization of rhythms across different frequency bands and biological substrates. For example, phase–amplitude coupling between gamma and theta is necessary for the normal attention process [39] and spike-phase coupling (Fig. 3d) among single spike firing and theta phase is important for coordination and adaptation [40]. Intriguingly, the brain–heart coupling, which is a hybrid interaction of different substrates, contributes to the brain's neural activity associated with the body's ‘neural subjective frame’ [41] and conscious functions [41,42]. In summary, the brain functions effectively in heterogeneous conditions at various levels, which requires quantities of qualitatively distinct nature to facilitate coherent information flow. The concept and construction methods of HNNs reflect these characteristics to a certain extent.

## THE FRAMEWORK FOR BUILDING HNNs

ANNs and SNNs exhibit substantial heterogeneity and distinct characteristics. Combining both paradigms to form HNNs endows a wide range of flexibility and diversity. Nonetheless, the distinct features and computing principles of each also pose considerable challenges to the construction of HNNs. For instance, ANNs and SNNs have different signal representations and information-processing characteristics, which should be addressed before cross-paradigm integration. Moreover, vertically bridging the gap between low-level structures and high-level functions of HNNs is also crucial. Therefore, effectively building HNNs requires comprehensive and systematic consideration from various facets, encompassing integration paradigms, fundamental theories, information flow, interaction modes and network structures.

### Integration paradigm

Constructing HNNs by integrating different neural networks requires deliberation on the appropriate coupling and integration direction to improve effectiveness. Two primary types of coupling are employed: tight coupling and weak coupling. Tight coupling occurs at a small scale and granularity, typically involving neuron models, basic circuits or modules. While it fosters novel basic models for HNNs, tight coupling may also complicate signal conversion due to frequent transformations between heterogeneous models. These HNNs integrate heterogeneous networks at a fine-grained level, presenting a vast design space. Such HNNs can be constructed using innovative hybrid neuron models and can be represented by the tuple $( {{{h}_\theta },G} )$ where ${{h}_\theta }$ represents the hybrid neuron model and *G* denotes a graph that describes the connection structure among these hybrid neuron models. These hybrid neuron models are characterized by possessing spatio-temporal dynamics and mixed-precision representation.

In contrast, weak coupling involves integrating heterogeneous models at the network level. In this approach, network interactions and signal conversions are less frequent but more concentrated. Hence, the constituent networks can be developed by adopting their respective construction technologies, employing a unified interface model for signal conversion. The framework proposed for the general design and computation of HNNs in [24] adopts this approach by first decoupling and subsequently integrating to construct hybrid multi-network models. To address connection challenges between different neural networks, a parameterized hybrid unit (HU) is introduced, which can be configured through expert-guided manual design or data-driven automatic learning. In the automatic learning scenario, HUs can be independently trained with specific objectives or jointly trained with connected heterogeneous networks, demonstrating great adaptability. This integration strategy combines the distinctive features of various heterogeneous computing paradigms while providing decoupling to enhance flexibility and efficiency.

In this scenario, the HNN can be succinctly represented by a tuple $( {{{F}_\theta },{{F}_\phi },{{H}_\psi },G} )$, where ${{F}_\theta }$ represents the set of the constituent ANNs and ${{F}_\phi }$ denotes the set of constituent SNNs. Essentially, ${{F}_\theta }$ and ${{F}_\phi }$ encompass various network architectures belonging to their respective paradigms. ${{H}_\psi }$ denotes the required HUs that bridge these heterogeneous networks together. *G* delineates a graph that describes the connection structure between these diverse networks within the entire HNN. The configuration of these four aforementioned components collectively shapes the design space of this particular type of HNN.

In general, weak coupling is more suitable when the basic ANNs and SNNs that constitute HNNs have relatively complete and independent functionality. This is particularly applicable when each network can be effectively optimized using mature methods from their respective domains. Conversely, tight coupling is more suitable for the opposite scenarios in which fine-grained integration is essential. Tight coupling enables a deep integration of the distinctive characteristics of both ANNs and SNNs, allowing the construction of new basic neuron models and expanding the research scope.

Heterogeneous integration can occur at corresponding or non-corresponding levels. Corresponding-level integration involves combining components at the same tier from two paradigms, such as ANN and SNN models, along with their respective algorithms. For instance, the hybrid plasticity algorithm [43] combines the error-driven global learning commonly used by ANNs with the biological synaptic plasticity mechanism of SNNs, enhancing abilities in continual learning and few-sample learning. Similarly, the neuromodulator-assisted credit assignment algorithm [44] incorporates a type of global neuromodulation mechanism into ANNs and SNNs for adjusting their synaptic plasticity, demonstrating improved recognition accuracy and continual learning capabilities with reduced computational cost. On the other hand, the spiking neural unit [45] incorporates the neural dynamics of SNNs into recurrent ANNs, enabling model integration. This approach promotes energy-efficient neuromorphic hardware implementations and synergistic neuroscience modeling.

Beyond corresponding-level integration, the two paradigms can also be integrated at non-corresponding levels. Integrating SNN algorithms into ANN models is feasible by introducing the plasticity mechanism of SNNs into the training of ANN models, or vice versa. Extensive literature supports this type of integration, allowing a combination of various model design techniques and training algorithms of deep-learning-based ANNs with SNN models [46–51]. This approach has significantly advanced models within their respective paradigms.

### Fundamental theory

To gain a comprehensive understanding of HNNs and their capabilities, it is essential to explore the fundamental theories associated with integrating paradigms from different aspects, including the theory underpinning signal representation and general transformation within heterogeneous models, the computational power theory of HNNs and the theory of generalization within the context of HNNs. The integration of heterogeneous models necessitates a rigorous definition of signal representation for each model to address the challenges posed by transformations, while general transformation models and methods are required to effectively expedite the construction of HNNs. The reported HU model [24] featuring intermediate representations provides evidence supporting the universality of the transformation model. These findings contribute to a deeper understanding of HNNs and facilitate advancements within this field.

The assessment of the upper limits of computational power in HNNs can provide valuable insights into the inherent universality of HNNs, thereby expanding their potential scope of applications. Furthermore, an examination of the generalization ability and computational learning theory of HNNs, considering specific network sizes and training data sets, can greatly facilitate the process of optimal hyperparameter selection for HNNs. These theories merit in-depth investigation to unlock their full potential.

### Information flow

Information flow is crucial for the effective functioning of HNNs, which encompasses various aspects including input and output locations as well as the contents being transmitted. To ensure a seamless information flow between networks, it is necessary to adjust the variables of the target network. Neural network variables can generally be divided into two groups: parameter variables (e.g. synaptic weights) and state variables (e.g. neuronal activation). Parameters evolve slowly over time, whereas states undergo frequent changes. From a systemic perspective, the manipulation and alternation of states enable information flow, while parameters are usually inaccessible. Notably, the classification of parameters and states is relative and can vary based on system settings and the perspective of the investigation.

**Figure 4.**
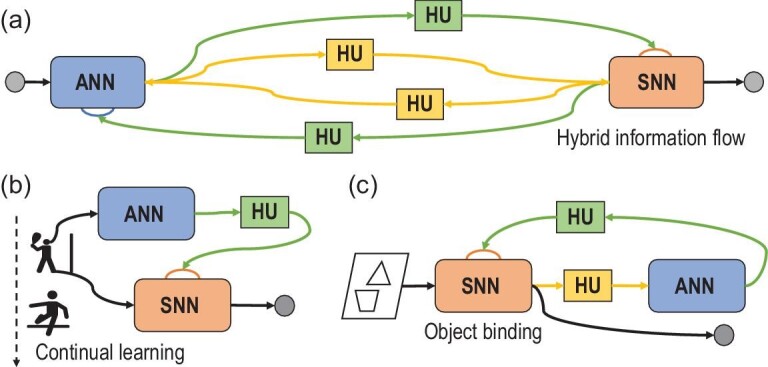
Hybrid information modulation and representative HNNs with hybrid modulation. (a) An illustration of hybrid information flow [24]. The transformation of hybrid information is facilitated by various HUs. (b) The hybrid modulation network [24] realizes a dynamic subnetwork allocation strategy based on task-level features, improving parameter reuse between similar tasks and alleviating catastrophic forgetting in continual learning. (c) The hybrid binding network [52] solves the ANN binding problem by combining SNN spike timing with reconstructive attention, resulting in a flexible, hierarchical and explainable representation that can bind multiple objects at different synchronous firing times.

Due to the distinct characteristics of states and parameters, the effects of applying actions to them vary. Building on the analysis above, information flow can be broadly categorized into two fundamental forms based on the input location: transmission and modulation. Information transmission refers to the output of one network affecting the state of another network, while information modulation involves the output of a network influencing the parameters of another network. Information transmission has an immediate and direct effect, while the effect of information modulation is relatively indirect and lasts for a longer time. Information transmission can be likened to the splicing of water pipes, exhibiting an additive effect, while information modulation is like controlling valves along these water pipes, manifesting a multiplicative effect. Moreover, as depicted in Fig. 4, hybrid information flows offer diverse spatio-temporal scales and employ rich coding schemes, allowing more flexible configurations of HNNs [24] and enabling various functions such as attention and resource scheduling.

Furthermore, the regulation of information flow can be achieved through a centralized network that can coordinate multiple networks. A method to accomplish complex tasks and efficiently coordinate ANNs and SNNs lies in the use of a hybrid neural state machine (HNSM)[53]. An HNSM, designed and built based on the connection structure of digital logic circuits, utilizes spiking neurons as its basic units. This neuro-based state machine controls both information flow and workflow within HNNs, providing promising advancements in control logic for such systems. For instance, the hybrid neural state tracker [54] applies the HNSM to high-speed tracking tasks by combining ANN-based detection with kernelized correlation filter tracking, demonstrating a significant enhancement in both tracking accuracy and speed.

Besides the state variables, the network can also transmit parameters to enhance the information output. For example, the transmitted information can be categorized into two types: original quantity and variations of the quantity, including temporal variations or spatial gradients. These diverse forms of information flow broaden the design possibilities for HNNs, thereby expanding the potential for complex functions.

### Interaction mode

The components constituting HNNs engage in various types of interactions, which can be analysed from an optimization perspective. Consequently, these network interactions can be classified as either collaborative or confrontational, depending on their impact on a specific objective function during training. When two networks align in their pursuit of optimizing a particular objective function, their relationship is deemed collaborative. Conversely, if two networks disagree on their optimization of a specific objective function, their relationship is considered confrontational. The interaction mode between networks is determined once the objective function is defined. For instance, Spike-GAN [55] introduces a hybrid architecture that combines an SNN-based generator and an ANN-based discriminator. The ANN guides the updating of the SNN using an adversarial learning strategy, enabling the network to effectively model the distribution of spiking signals.

During the training of a multi-network model, the presence of multiple objective functions can coexist, leading to both collaborative and confrontational relationships between networks. In multistage training, these relationships can occur sequentially. The presence of uncertain objective functions can result in intricate interactions among networks. Moreover, the interactions between multiple networks within a single network yield mutual effects that propagate through information flow and subsequently influence other networks. These interactions not only expand the design possibilities and flexibility of multiple network models, but also lay the groundwork for developing advanced intelligence.

### Structure and function

**Figure 5.**
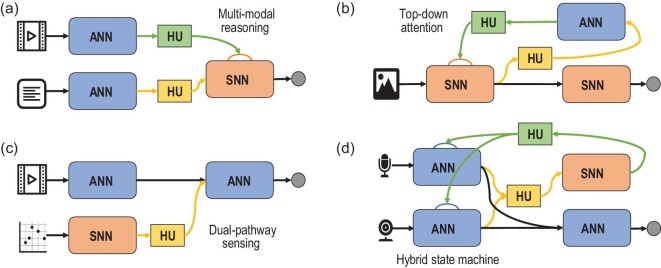
Representative HNNs with different structures. (a) The hybrid reasoning network [24] with serial structure for multistage robust question answering. (b) The hybrid top-down attention network [56] with feedback structure for multilevel efficient perception. (c) Hybrid sensing network [24] with parallel structure for multipathway tracking. (d) An HNN-based self-driving system [2,57] with composite structure for simultaneously realizing real-time object detection, tracking, voice control, obstacle avoidance and balance control.

Based on the analysis of information flow and interaction modes, high-level functions can be achieved by carefully designing the structure of HNNs. When considering an HNN as a directed graph, its basic topological connectivity can be classified into three categories: serial, parallel and feedback structures, as illustrated in Fig. 5. The serial structure proves particularly beneficial for the multistage processing of data from the same source. By selecting appropriate networks for processing at different stages based on data characteristics and task requirements, the serial configuration enables efficient data processing. An example is the hybrid reasoning network [24] that implements a full-network neuro-symbolic system, which utilizes heterogeneous transmission for interpretable, robust and parallel multimodal reasoning. Moreover, the effectiveness of the serial structure is illustrated in visual place recognition tasks, as evidenced by the application of a compact yet highly performant HNN [58].

Parallel structures are highly suitable for processing multimodal data sources by simultaneously utilizing multiple networks. Due to the inherent dissimilarities in data sources, which entail different symmetries and necessitate distinct representation structures and operations, utilization of various network modules tailored to each type of data becomes a compelling necessity. For example, visual cameras and laser radars provide distinctive information about a given target, therefore requiring specific types of networks tailored to their specific data characteristics. An excellent illustration of this concept is the hybrid sensing network [24], which implements a parallel structure with heterogeneous transmission paths. This network enables multipathway sensing, resulting in exceptional high-speed tracking capabilities with an impressive frame rate of 5952 while maintaining satisfactory accuracy. The reason why this HNN achieves such performance is through the combination of the high-speed and energy-efficient nature of SNNs with the high precision of ANNs, resulting in a better overall performance balance.

Feedback structures offer the advantage of concurrent multilevel information integration, wherein data with varying processing levels synergistically contribute to enhancing the adaptive capabilities of the overall system. Specifically, in the context of visual processing, high-level information obtained through feature extraction can effectively regulate the operations of the front-end network. Evidencing the principle of feedback structures is the hybrid top-down attention network [56], which combines a feedforward SNN and a feedback ANN to effectuate a form of top-down attention mechanism. The ANN generates attention maps based on extracted features from the SNN, thereby modulating the encoding layer within the SNN. Through the attention maps, the SNN channelizes its processing resources towards the most informative sensory inputs. This model showcases remarkable robustness against adversarial noise, high computational efficiency and improved interpretability. The firing rates of the HNNs are significantly reduced by ≤50% compared with their corresponding SNN counterparts, thanks to the adaptive processing capability enabled using the hybrid modulation technique.

A multi-network motif can be created by combining assorted topologies, information flows and network interactions, leading to the development of advanced functions. As mentioned earlier, in multilevel collaborative networks, adaptive information processing such as top-down attention mechanisms can be achieved through information modulation with feedback structure. These multi-network motifs can be further merged to form large-scale multi-network models with composite structures capable of accommodating complex functions. Moreover, the connection structure of HNNs can undergo dynamic alterations based on input features or external modulation signals, enabling capability with enhanced adaptive processing and holding substantial merits for further investigation.

### Applications of HNNs

Through the aforementioned design dimensions, the construction of flexible and adaptable HNN models becomes feasible. This paper presents three promising application directions for harnessing the advantages of HNNs: utilizing data heterogeneity, utilizing hardware heterogeneity and neuroscience modeling. The utilization of data heterogeneity refers to the varying nature of data processed across different stages or paths, classified as parallel and serial data heterogeneity. It is worth noting that these data heterogeneities are highly correlated with the structure of HNNs.

Serial data heterogeneity denotes significant variations in data characteristics during processing, necessitating the use of different networks for effective data processing. An example is the Spike-FlowNet [59], which integrates SNNs and ANNs in a sequential structure. This integration enables efficient estimation of optical flow from sparse asynchronous event camera outputs without compromising performance. Furthermore, in a brain–computer interface (BCI) system, spike signals or electroencephalogram data can be initially recorded and processed using SNNs. Subsequently, an ANN-based feature extractor is utilized to conduct an in-depth analysis [60]. Due to the analogy between BCI and HNNs, it is natural to explore HNNs in BCI scenarios.

Parallel data heterogeneity refers to the ability to capture different types of data from the same object using different sensors or preprocessing operations, which are subsequently processed concurrently by different networks. Integrating multiple heterogeneous data sources generates parallel data heterogeneity. An example is the Dynamic and Active-pixel Vision Sensor that combines both frame-based cameras and event cameras, thereby yielding a heterogeneous data set that is suitable for processing through HNNs. Notably, the hybrid sensing network [24] has been reported as adept at handling the heterogeneous data produced by multi-output sensors. Furthermore, the acquisition of heterogeneous data using multiple preprocessing operations is elucidated through the hybrid modulation network [24], which employs a hierarchical feedforward structure with diverse modulation schemes to achieve hierarchical abstraction of task information. This hierarchical approach enhances parameter reuse across similar tasks and mitigates the occurrence of catastrophic forgetting in continual learning scenarios. After learning 40 tasks, the HNN exhibits a notable improvement in mean accuracy across these tasks by ∼50% in comparison with the single SNN baseline. This remarkable advancement is made achievable through the dynamic allocation of resources, which is guided by the specific characteristics of the task and facilitated using the innovative hybrid modulation technique. In conclusion, HNNs can effectively integrate the respective advantages of different networks in situations involving data heterogeneity, enabling enhanced information-processing capabilities.

In addition to its information-processing capabilities, HNNs offer notable advantages in terms of energy efficiency. To optimally leverage these energy-efficient benefits, the development of hybrid chips through digital–analog hybrid circuits is crucial and promising. This implementation approach is particularly suitable for robot control applications that necessitate a balance between real-time operation, power consumption and accuracy.

Pioneering this field, an HNN-based self-driving system [2,57] has been implemented using a Tianjic chip that showcases the simultaneous processing of versatile models, thereby enabling real-time object detection, tracking, voice control, obstacle avoidance and balance control. To facilitate flexible collaboration within hybrid networks, the system employs rich coding schemes and a trainable HNSM. A memristor-based HNSM [61] has also been reported that exhibits the capacity to handle non-ideal behaviors with strong robustness, while concurrently benefitting from these irregularities to exhibit accelerated convergence during training. These hardware implementations successfully strike a better balance between energy efficiency and high performance.

Neuroscience modeling is another promising application that can harness the advantages of HNNs. Recently, an emerging transition in neuroscience modeling is to exploit a deep-learning framework to account for more complicated neural functions. This endeavor entails the establishment of an analysis pipeline that links the real-valued vector representation in deep networks and the functional magnetic resonance imaging (fMRI) and electroencephalography (EEG) signals observed in the brain. However, the deep-learning framework has inherent limitations when it comes to accommodating spike-level representations, such as spike-timing codes and synchrony. Consequently, the analytical approach tends to be mainly confined to the mean-field comparison, overlooking the fine-grained details of spike-level interactions.

**Figure 6.**
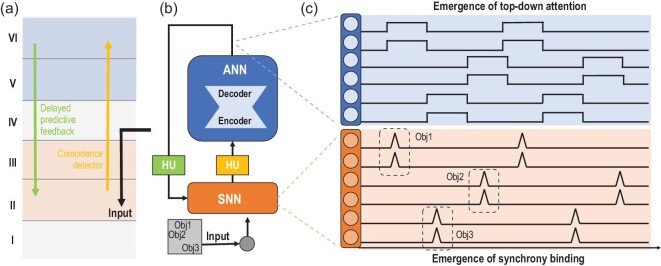
An instance of modeling the structure, function and behavior of the neocortex with HNNs. (a) A simplified sketch of the cortical column, highlighting the bottom-up coincidence filtering and delayed predictive top-down feedback, which inspire the design of HUs in the HNN. (b) The HNN establishes a processing loop, incorporating both bottom-up and top-down mechanisms, between an SNN and an auto-encoder. In this process, the coincidence detector (the bottom-up HU in (b)) integrate spikes within a narrow time window to the encoder (the ANN in (b), abstracting away the non-linearity in the bottom-up pathway in (a)). The output of the decoder (the ANN in (b), abstracting away the non-linearity in the top-down pathway in (a)) undergoes a delay (the top-down HU in (b)) before modulating the spiking neurons. (c) Through iterative dynamics between the ANN and the SNN, the spiking neurons in the SNN dynamically form a synchrony pattern that binds the features of multiple objects (bottom) while switching top-down attention emerges in the delayed feedback of the decoder output (top).

On the contrary, the HNN framework naturally lends itself to the simulation of cross-scale interaction among observables including mean-field activity for modulation and precise spike synchrony for coding and transmission, as presented in Fig. 6. In a recent HNN model [52], a cortical-inspired architecture is built in which the top-down mean-field attention is modeled using an ANN while the bottom-up coincidence filtering is modeled using an SNN. The iterative interaction between the top-down ANN and the bottom-up SNN leads to the emergence of synchrony coding patterns in the SNN, which is comparable to a wide range of cortical phenomena [62–64] and provides a bio-plausible solution to the fundamental binding problem in neuroscience [65–67]. HNN modeling provides the basis for efficiently modeling both fast spike-timing synchrony and slow mean-field oscillation concurrently, which can be achieved through generative autoregressive mechanisms in ANNs.

## THE PLATFORM FOR SUPPORTING HNNs

The widely acknowledged consensus is that graphic processing units have played a pivotal role in the recent rapid advancements of artificial intelligence, due to their large number of cores and high-speed memory that enable proficient parallel computing. Consequently, to facilitate the efficient deployment and applications of HNNs, the development of appropriate supporting systems becomes imperative. These platforms encompass a range of components, including chips, software and system infrastructure. Below, we outline the notable advancements achieved in these fields.

### Chip

Historically, different computing paradigms have typically aligned with specific hardware platforms. The emergence of dual-brain driven and multi-network integration has generated a compelling demand for developing general computing platforms to efficiently implement diverse neural models and algorithms. However, the fundamental differences in the formulations and coding schemes between computer-science-oriented models and neuroscience-oriented models make this task challenging. The Tianjic [2] BIC chip stands as the pioneering cross-paradigm chip capable of simultaneously and efficiently supporting ANNs, SNNs and HNNs. This has been facilitated by the design of a unified model description framework that caters to hybrid computing paradigms, leading to substantial enhancements in power-utilization efficiency and throughput [68]. Moreover, Tianjic strikes a balance between performance and resource costs during the mapping [69] and deployment process [70]. The more recent iteration, TianjicX [31], has further enhanced programmability and incorporated a brain-inspired-complete coarse-grained primitive set to support versatile algorithms. Notably, the design of TianjicX introduces temporal flexibility atop the spatial parallel architecture of traditional neuromorphic chips, thereby enabling the fusion of control-flow and data-flow architectures. This spatio-temporal elasticity facilitates optimal and flexible utilization of the computing resources of BIC chips.

Currently, several leading BIC chips have adopted similar design philosophies by introducing support for cross-paradigm modeling. Representative examples are Intel Loihi 2, SpiNNaker 2 and BrainScaleS 2. According to the technology brief [71], Loihi 2 can support both binary-valued spike messages and integer-valued payloads for graded spikes. SpiNNaker 2 [72] introduces a hybrid computation approach that facilitates the simultaneous implementation of ANNs and SNNs by adding a dedicated multiply-accumulate (MAC) array for intensive matrix operations. BrainScaleS 2 [73] highlights underlying support for mixed-precision representation and hybrid plasticity, thereby enabling simultaneous support for both backpropagation training of ANNs and local learning of SNNs. Shenjing [74], NEBULA [75] and an advanced neuromorphic chip in TCAS [76] have likewise incorporated optimizations for cross-paradigm models in their architectures. Beyond chips, IBM's computing-in-memory devices [45] have also introduced cross-paradigm design. In conclusion, hybrid computing establishes a new approach and perspective for intelligent hardware design, which allows comprehensive integration and complementary utilization of hardware technologies from various domains, thereby effectively leveraging their individual strengths.

### Software

System software functions as the intermediary interface connecting applications and hardware. In conventional BIC paradigms, software acts more like a tool for facilitating efficient modeling and optimally leveraging hardware resources. In the initial stage of software development for HNNs, it inherits the modeling and deployment functionalities from conventional BIC software. Recent efforts have been directed towards accommodating the dual modeling requirements of ANNs and SNNs. For instance, Spyketorch [77] and SpikingJelly [78] expand SNN operators and coding rules from the ANN programming framework PyTorch [79]. Building upon the ANN programming framework Keras [80], the Loihi group has developed the deep SNN modeling framework NxTF [81] with learning capabilities. Similarly, the Tianjic group has introduced a programming framework [82] based on PyTorch for flexible modeling of HNNs with various HUs. This framework also supports versatile precision conversion through automatic quantization techniques. Its newly reported deployment platform, BiMap [83], explores the compilation of HNNs on a many-core architecture.

In the subsequent development stage, the co-evolution of software and hardware is emphasized to form a comprehensive HNN system. The software architecture is tailored to encapsulate the fundamental aspects of HNNs and formalize distinctive attributes of HNNs through corresponding computational models. Notably, a BIC software hierarchy is reported that combines precise computing and approximation with neuromorphic completeness [32,84]. It relaxes requirements for hardware and improves compatibility for various programs, facilitating the development of general-purpose neuromorphic applications. The runtime abstractions offered by TianjicX [31] integrate control-flow and data-flow execution patterns, enabling mutual scheduling capabilities.

Furthermore, the HU proposed in the HNN framework implicates the efficient and frequent transformation of data or control protocols during runtime. This capability enables seamless interconnectivity across multiple heterogeneous subsystems. In conventional compiling techniques, runtime transformations between different execution or language systems are achieved via Just-In-Time (JIT) compilers. With the HNN hardware, the concept of embedding JIT techniques among HUs can emerge in the future, which gives rise to the development of hyper-heterogeneous systems.

### System infrastructure

To facilitate the execution of HNNs, a robust system infrastructure is necessary to enable multilevel, multi-granularity and large-scale cross-paradigm integration. This infrastructure encompasses critical components such as data storage, communication infrastructure and resource scheduling. Until now, significant progress has been made in both small and large-scale system implementations within this field [18,71,85–91].

SNNs and ANNs have distinct training and distribution requirements. For the large-scale training and distribution of HNNs, it is necessary to employ a heterogeneous hierarchical brain-inspired architecture. In ANNs, neurons communicate by using high-precision and continuous-value-encoded activations, transmitting information mainly in the spatial domain (across layers). On the other hand, communication among neurons in SNNs occurs through binary events rather than continuous activation values, resulting in asynchronous updates of state variables. Consequently, asynchronous non-blocking communication is utilized to handle different time steps within a single batch, whereas synchronous blocking mode is employed for gradient updates across different batches. Developing various pipelining designs based on time/event-driven approaches is important for satisfying the time-step and batch-split pipelining needs. In scenarios necessitating high precision over multiple time steps, a data update scheme based on ‘time-driven’ pipelining is required. Conversely, an optimization scheme based on ‘event-driven’ batch pipelining is more suitable for handling large data sets. In summary, novel interconnection networks featuring flattened and event-driven features, along with hardware-accelerated communication protocols, are needed to address the demands of HNNs within large-scale brain-inspired clusters.

**Figure 7.**
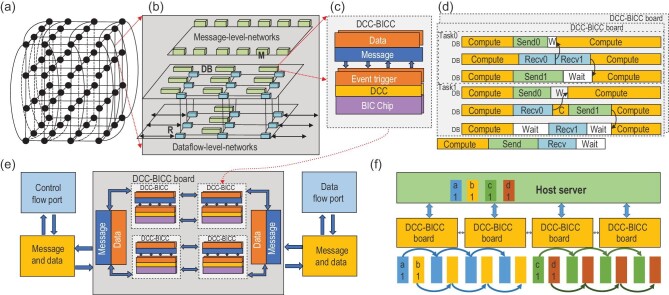
A system infrastructure of HNNs. (a) The interconnection structure between nodes in the BIC platform utilizes a 3D-torus topology to facilitate seamless communication in both horizontal and vertical dimensions. (b) The data network facilitates efficient data communication through a 3D-mesh topology, while the message network ensures smooth control communication using a ring topology. This design strategy is advantageous in reducing communication latency and enhancing parallelism. (c) Structure of the distributed configurable controller (DCC) and brain-inspired computing chip (BICC). The DCC plays a key role in supporting hardware interconnectivity and communication functionalities, while the BICC is a crucial component for implementing computations. (d) Synchronization mechanism of DCCs and BICCs. (e) DCCs and their communication structure. The control-flow port enables the integration communication of data at different scales between host computers and BIC nodes. The data-flow port enables large-scale expansion of BIC nodes. The distributed semaphore mechanism coordinates the control, storage and synchronization of the system. (f) An illustration of the scatter primitive that coordinates the communication between the server and the BIC chips based on the HNN system infrastructure.

The Jingwei-2 cluster (Fig. 7) is a technical solution designed for brain-inspired multi-network systems. This cluster introduces pioneering architectural enhancements in the configuration of a hard cluster, incorporating advancements throughout the entire design stack to address interconnection requirements in lateral, vertical and horizontal dimensions. Several key features are incorporated including a configurable controller, communication primitives catering to the data-flow patterns of the intelligent computing system as well as virtualization components. Its primary goal is to address challenges related to hardware interconnection, data communication and computational processing in BIC systems. Efficient BIC cluster systems will enhance scalability and heterogeneous fusion, thereby facilitating the construction of larger integrated systems. This advancement will open up broader avenues for research across diverse possibilities in various fields such as medicine and neuroscience, and accelerate a broader range of applications.

## SUMMARY AND OUTLOOK

The concept of a dual-brain-driven paradigm has gained increasing attention in the field of BIC due to its potential implications for the advancement of AGI. One manifestation of this paradigm is represented by HNNs, which adopt principles from both neuroscience and computer-science disciplines. This paper provides a systematic review of HNNs, including their origin, biological perspective, construction methods, chip, software and supporting system infrastructure. Subsequent deliberations focus on potential research directions and subjects of HNNs.

### Construction framework

SNNs are important constituent networks of HNNs. Despite the recent rapid progress, research on SNNs has yet to keep pace with that on ANNs. The unique information-processing capabilities of SNNs and efficient hardware implementation strategies deserve further investigation so that the strengths of integrating ANNs and SNNs can be optimally leveraged.

Fundamental theories associated with the computational power and generalization of HNNs are important for constructing HNNs and practical applications. However, the literature on this subject remains relatively sparse. Drawing insights from both machine-learning theory and neuroscience could facilitate the advancement of HNN theories.

The modes of integration in HNNs influence their performance. Confrontation between constituent networks holds the promise to improve the stability and robustness of HNNs. Incorporating principles from adversarial learning in deep-learning fields is anticipated to facilitate the development process.

Dynamic connections in HNNs merit further research attention, owning to their potential to enhance adaptability and flexibility. Furthermore, the integration of tightly coupled HNNs at a small granularity presents an auspicious avenue for constructing novel basic modules that support the development of AGI.

Assessment metrics for HNNs have not been thoroughly investigated, highlighting a promising avenue for evaluating and improving HNNs. For instance, with regard to implementation, it is essential to explore a comprehensive deployment evaluation framework that takes into account latency, power consumption and other relevant factors. This will offer tangible guidance for efficient implementation of HNNs. In terms of functionality, delving into the reliability and robustness of heterogeneous information transformation in HNNs will aid in debugging models and expediting model development.

### Promising applications

HNNs provide a unique platform for exploring AGI. For instance, as embodied artificial intelligence [92] emerges, intelligent agents need to interact with the environment through various sensory channels such as vision, hearing and touch, while possessing a physical body. These perception tasks, closely tied to sensor capabilities, require versatile basic models intrinsic to HNNs due to their ability to meet diverse performance and cost requirements. Furthermore, SNNs nested within HNNs, equipped with intricate state memory and spatio-temporal dynamics, offer promising substrates to effectively handle continuous interactions with the environment. Consequently, a deeper exploration of these networks holds promise in unraveling profound insights.

Research on large-scale HNNs is of utmost importance. In the domain of deep learning, transformer-based deep models have been extensively scaled to possess billions of parameters and have undergone pre-training on massive data sets. These advancements have showcased remarkable capabilities in natural language processing and image understanding. However, the scale of existing HNNs remains relatively modest and the available training data are not sufficient. Hence, it will be highly valuable to delve into further research concerning the design, optimization and availability of extensive training data for large-scale HNNs.

### Supporting platforms

On another note, hybrid computing platforms require further development to enable the training and deployment of large-scale HNNs. Key components of the system-level platform including computing chips, communication networks and memory hierarchy necessitate continual co-evolution with HNNs. Large-scale HNNs supported by such platforms not only facilitate the development of embodied AGI, but also serve as building blocks for constructing brain-inspired foundation models that possess both similar intelligence and structure to the human brain. Beyond the general intelligence characteristic of current foundation models [29,30], these brain-inspired foundation models can function as artificial brains for investigating human intelligence, thereby promoting the synergistic advancement of brain science and artificial intelligence.
